# Impact of educational interventions on primary prevention of cardiovascular disease: A systematic review with a focus on physical activity

**DOI:** 10.1080/13814788.2017.1284791

**Published:** 2017-03-08

**Authors:** Ana Ramôa Castro, Nórton L. Oliveira, Fernando Ribeiro, José Oliveira

**Affiliations:** ^a^ Espaço Saúde Health UnitPortoPortugal; ^b^ Research Center in Physical Activity, Health and Leisure, Faculty of Sport, University of PortoPortoPortugal; ^c^ School of Health Sciences and Institute of Biomedicine – iBiMED, University of AveiroAveiroPortugal

**Keywords:** Systematic reviews and meta-analyses, health education, prevention, general practice/family medicine, general

## Abstract

**Background:** Evidence from epidemiological and experimental studies illustrates the beneficial impact of healthy lifestyle behaviours on cardiovascular risk.

**Objectives:** To assess the effectiveness of primary care health education interventions designed to promote healthy lifestyles on physical activity levels and cardiovascular risk.

**Methods**: A computer-aided search on PubMed and Scopus was performed to identify relevant studies published from January 2000 to October 2016. Two authors independently selected studies for inclusion and extracted data, including intervention characteristics and outcome measures, namely physical activity and cardiovascular risk or risk factors.

**Results:** Of the 212 identified studies, 15 met the inclusion criteria. The 15 studies enrolled 6727 participants; the sample size varied between 74 and 878 adults. Fourteen studies assessed physical activity by questionnaire and only one study used accelerometry. Eight of the 15 studies showed improvements in the physical activity levels after the intervention, ranging from 5% to 26% in those where significant changes between groups were detected. Most studies reported significant positive effects of the health education interventions on cardiovascular risk factors, mainly on lipid profile, blood pressure and cardiovascular risk score.

**Conclusion:** The health education interventions, in primary care, seem to improve daily physical activity, cardiovascular risk factors and risk score.

KEY MESSAGES

- Health education interventions increase daily physical activity levels.
- Primary care interventions focusing on healthy lifestyles improve cardiovascular risk score, lipid profile and blood pressure

## Introduction

The mortality attributed to cardiovascular diseases (CVD) has fallen considerably in the last decades; nonetheless, it remains the major cause of premature death in Europe and worldwide [1]. The most recommended management strategy to reduce cardiovascular risk and cope with modifiable cardiovascular risk factors, including sedentary behaviour, overweight/obesity and hypertension [1], is the change of unhealthy lifestyle behaviours [2–5]. Primary healthcare interventions, by preventing and modifying CVDs risk factors [6], are a frontline strategy to fulfil this purpose. However, many barriers hamper the implementation of the recommended ‘high-risk’ approach, such as health professionals’ difficulties to assimilate multiple risk factors into an accurate assessment of cardiovascular risk [7]. Indeed, the adherence to the guidelines and lifestyle counselling is less than optimal and often abandoned by primary caregivers [7,8].

Lifestyle and health education programmes tend to be multidisciplinary with self-care components tailored to individual risk factors [9]. The beneficial impact of healthy lifestyle behaviours on cardiovascular risk was demonstrated in a three-year randomized trial in the primary care setting [10].

Health education refers to the improvement of individual, group, institutional, community and systemic strategies to expand health knowledge, attitudes, skills and behaviours [11]. It aims to enhance health literacy, and behaviour and lifestyles changes conducive to health through the educational process [11]. Usually, these programmes use different channels (mobile applications, face-to-face, text messaging, internet based tools, written educational materials such as flyers and booklets) to empower individuals to adopt healthy lifestyles [11].

Previous reviews on this topic [2–5,12], focusing on a specific tool to deliver the intervention (e.g. text messaging, face-to-face interventions), were conducted. In contrast, the present review is broader by not focusing on intervention tools but by highlighting the intervention features (intervention design) that make it most likely to increase daily physical activity (PA). In the present review, we provide a critical review of the literature linking healthy lifestyles, cardiovascular risk and/or risk factors and PA in primary care, and to discuss the impact of those interventions on PA. Having in mind the importance of PA, the aim of this review is to analyse the effectiveness of health education interventions for change of lifestyle, with particular emphasis on PA and cardiovascular risk, in primary care.

## Methods

### Databases and search strategy

We used preferred reporting items for systematic reviews and meta-analysis statement (PRISMA) standards to systematically search PubMed and Scopus for studies in the English language that evaluated the effects of health education interventions focused on change of lifestyle in primary care, on humans, published from January 2000 to October 2016 [13]. The search terms were: (counselling OR education OR intervention OR health promotion) AND (primary care) AND (cardiovascular risk) AND (physical activity OR healthy lifestyles) AND (randomized controlled trials). Reference lists of studies identified by electronic searches were then searched to identify further articles relating to the topic of the review and to ensure that appropriate articles were obtained. In addition, to avoid retrieval bias, we manually searched the reference lists of landmark studies and background articles on this topic to look for any relevant citations that electronic searches might have missed.

### Selection criteria for studies

All studies retrieved from our search needed to meet the following inclusion criteria: randomized controlled trials; adult human subjects in primary prevention; submitted to a health education intervention, as defined in the introduction, aiming to enhance health literacy, and the adoption of healthy lifestyles; with PA as endpoint, regardless of being the primary or secondary endpoint. Studies were excluded on the basis of the following: review papers; letters or editorial articles; studies not involving a health education intervention; studies involving interventions with supervised exercise sessions; studies with children and adolescents and those only involving individuals aged over 65 years.

### Study selection

**Figure 1.**
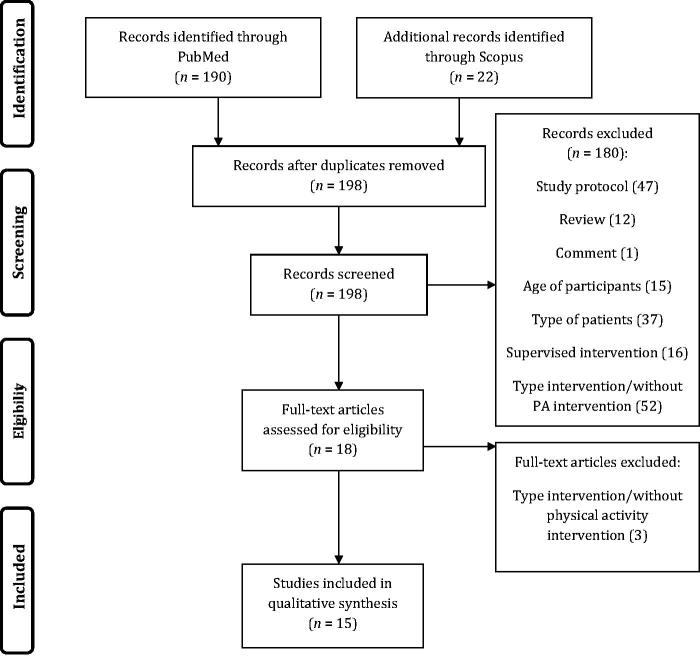
Study identification and selection flow diagram.

Two authors determined whether studies fulfilled the criteria for inclusion in this review through screening titles, abstracts, and keywords of the studies identified in the electronic search. When both authors failed to reach an agreement, the full text of the respective study was obtained and analysed to establish suitability. All studies classified as relevant by either of the authors were retrieved. Then, a standardized form was used to determine the eligibility for inclusion in the review based on the information within the full paper. A third author resolved disagreements. Figure 1 shows the process of the study identification and selection.

### Data extraction and methodological quality assessment

Two authors independently extracted data relevant to the review using a customized form. Data were extracted for study design, population, type and characteristics of the intervention, and outcomes, namely PA and cardiovascular risk or risk factors. Two authors using the PEDro scale independently assessed the methodological quality of each study. A third author resolved disagreements. The PEDro scale comprises 10 items that relate to internal validity and interpretability [14]. The scale provides an overall indication of RCT quality in a scale of 0 to 10. A study was rated as having ‘high’ methodological quality if it attained six points or more; the PEDro scale is a valid and reliable measure of the methodological quality of clinical trials [14,15].

## Results

A total of 212 studies were identified in the electronic databases search. After reviewing the title and abstract, 13 papers were excluded by being duplicates (i.e. coming from the two different databases). Of the remaining 198 papers, 184 were further discarded due to the following reasons: comprised supervised exercise interventions (*n* = 16), the studies did not assess a measure of PA and type of intervention (*n* = 56), enrolled participants with established disease (for instance ankylosing spondylitis) (*n* = 36), review papers (*n* = 12), comments (*n* = 1), participants were children or old adults (*n* = 15), or pregnant women (*n* = 1), papers describing the study protocol for a randomized controlled trial (*n* = 47) (Figure 1). Therefore, only 15 studies were included in this review (Table 1). One study was excluded from the review because it has two publications of the same study [16].

**Table 1.** Studies of the effects of health education intervention in physical activity.

Reference	PEDro score	Participants	Intervention		PA assessment	PA results/conclusions	
			Type/provider	Follow-up			
Azar et al., 2016 [17]	8/10	74 participants Immediate Group: 37 (22 women); 59.6 ± 11.9 years Delayed group: 37 (22 women); 59.8 ± 10.5 years	24 weekly face-to-face groups videoconference + seven in-person group based PA Physicians, nutritionists, exercise physiologists and lifestyle coaches	Six m	Stanford seven-day PA recall	Change from baseline to three m, mean (95% CI): Immediate group: 652.8 (236.8–1068.7) Delayed group: 103.6 (–294.8–501.9) Between-group difference: 549.2 (–26.8–1125.2)	No changes in PA in either group at three and six months
Griffin et al.,2014 [27]	8/10	478 participants with T2DM. IG: 239 (89 women), 59.5 ± 7.5 years CG: 239 (91 women), 59.8 ± 7.5 years	Intensive treatment plus a theory-based behaviour change intervention: 1 h introductory meeting + six 30 min meetings + four brief phone calls Lifestyle facilitators	12 m	Four days accelerometry and Norfolk PA questionnaire	IG: 90.0 ± 55.1–95.2 ± 55.7 MET- h/week CG: 78.6 ± 48.0 to 80.1 ± 49.5 MET-h/week	PA improved in both groups. No significant differences between groups at one year in PA both objectively and subjectively measured.
Reid et al., 2014 [29]	7/10	426 family members of patients with CAD. IG: 211 (128 women), 52.0 ± 11.9 years CG: 215 (133 women), 51.1 ± 11.3 years	17 counselling sessions (one face-to-face +16 telephone); weekly for the first 12 weeks and then at weeks 16, 20, 26, 39 and 52 Health educator	12 m	Modified Godin leisure-time exercise questionnaire	IG: 91.7 ± 102.5 to 142.5 ± 122.0 min/week CG: 88.7 ± 99.7 to 118.6 ± 109.1 min/week	The IG showed higher PA levels than the CG.
Lakerveld et al.,2013 [24]	8/10	622 adults at risk for T2DM and CVDs. IG: 314 (178 women), 43.6 ± 5.1 years CG: 308 (185 women), 43.4 ± 5.5 years	Cognitive behavioural program: six 30-min counselling sessions + three-monthly sessions by phone for one year + health brochures Practice nurses	12 m	SQUASH questionnaire	Values are median (Q1;Q3) of moderate PA: IG: 56 (19–150) to 52 (21–138) MET min/day CG: 47 (19–120) to 56 (26–126) MET min/day	No changes in PA
Hardcastle et al.,2013 [22]	7/10	334 participants with CVDs risk factors. IG: 203 (*n* of women n/a), 50.1 ± 0.7 years CG: 131, 50.4 ± 1.0 years	Standard exercise and nutrition information plus up to five face-to-face motivational interviewing sessions PA specialist, registered dietitian	Six m	IPAQ	Walking time: IG: 996.1 ± 1116.6 to 1195.54 ± 1277.6 METmin/week CG: 1242.5 ± 1432.7 to 1050.5 ± 1344.4 METmin/week	The IG significantly increased walking time.
Cochrane et al.,2012 [19]	8/10	601 participants with Framingham score ≥20%. IG:236 (32 women), 63.3 ± 6.4 years CG: 365 (36 women), 63.9 ± 6.5 years	NHS health check service + support for lifestyle change based on the motivational interview/counselling model Lifestyle coach	Support upto 12 m	General practice PA questionnaire	Mean PA score: IG: 2.07–2.81 CG: 2.65–2.80	PA scores improved in both the CG (NHS health check only group) and the IG (NHS health check plus additional lifestyle support)
Harris et al., 2012 [20]	7/10	699 participants either aged 56–64 years or 40–55 years with hypertension or dyslipidaemia. IG: 384 (232 women), mean age n/a CG: 315 (169 women)	Brief lifestyle advice and motivational counselling: one individual session + four 1.5-h sessions over the first three months and a further two follow-up sessions at six and nine months. GP, nurses and practice managers	Nine m	The brief PA assessment tool	PA score: IG: 3.71, 4.59 and 4.60 CG: 3.38, 3.89 and 4.09	PA increased to a greater extent in the IG both at six and nine months
Parra-Medina et al., 2011 [8]	9/10	266 women with hypertension or diabetes. IG: 136, mean age n/a CG: 130	Theory-based lifestyle intervention targeting PA and dietary fat intake: tailored telephone counselling and tailored newsletters Primary care providers and nurses	12 m	CHAMPS Questionnaire	Odds ratios (95%CI) of increasing leisure-time moderate-to-vigorous PA: IG: 3.82 (1.41, 10.30) (at 6 months) 1.76 (0.62, 5.0) (at 12 months)	The IG showed higher total and leisure-time moderate-to-vigorous PA at 6 months but not at 12 months
Koelewijn-Van Loon et al., 2009 [23]	7/10	589 patients eligible for cardiovascular risk management. IG: 304 (174 women), 56 ± 10 years CG: 285 (151 women), 58 ± 10 years	2 face-to-face consultations (15-20 min) + 10 min telephone consultation (or a face-to face consultation) Nurses	12 w	CHAMPS Questionnaire	Moderate or vigorousintensity PA: IG: 405 ± 343 to 460 ± 362 min/week CG: 447 ± 345 to 449 ± 365 min/week	No changes in PA
Holmen et al.,2014 [28]	7/10	151 Diabetic participants FTA Group: 51 (17 women); 58.6 ± 11.8 years FTA-HC: 50 (25 women); 57.4 ± 12.1 years CG: 50 (20 women); 55.9 ± 12.2 years	FTA Group: mobile phone with self-management system FTA-HC group: five phone based conversations (20 min) during four months for health counselling with motivational interviewing based on the transtheoretical model. Nurses	12 m	HeiQ questionnaire	FTA group: 2.78 (2.52, 3.04) to 2.82 (2.60, 3.05) FTA-HC: 2.78 (2.57, 2.99) to 2.81 (2.57, 3.04) CG: 2.71 (2.51, 2.92) to 2.81 (2.58, 3.04)	No changes in PA
Armit et al., 2009 [25]	6/10	136 participants not meeting PA recommendations (82 women). IG (ES): 45 (31 women), mean age n/a IG (ES + P): 45 (27 women) CG: 46 (24 women)	ES: GP usual care +30 min PA counselling based on the transtheoretical model. ES + P: as for ES group, with goal setting (steps/day) and self-monitoring focusing on a pedometer GP and Exercise scientists	12 w (24 w follow-up)	Active Australia PA questionnaire	Odds ratios (95% CI) for meeting the National Physical Activity Guidelines IG (ES + P): 2.39 (1.01, 5.64) IG (ES): 1.14 (.47, 2.76)	PA improved in all groups. At week 24, the ES + P group were more likely to report meeting PA guidelines than the CG
Davies et al., 2008 [26]	7/10	824 patients with T2DM. IG: 437 (47 women), 59 years (SD n/a) CG: 387 43 women), 60 years	Six-hour structured group health education programme focused on lifestyle factors, such as food choices and PA Registered healthcare professionals	12 m	IPAQ	No IPAQ data is reported.	The IG showed greater increase in PA at four months
Wister et al., 2007 [9]	8/10	315 participants with a Framingham score ≥10% IG: 157 (86 women), 55.8 ± 5.5 years CG: 158 (98 women), 55.1 ± 5.2 years	Report card showing person’s risk profile + telehealth-guided self-care management system at every six months Lifestyle counsellors	12 m	Five-point ordinal scale	Difference between baseline and one year, mean (95% CI): IG: 0.17 (–0.06–0.40) CG: 0.16 (–0.08–0.40)	No differences between groups
Hardcastle et al., 2008 [21]	7/10	334 participants with CVDs risk factors IG: 203 (*n* of women n/a), 50.1 ± 0.7 years CG: 131, 50.4 ± 0.9 years	Patient-centred counselling intervention that incorporated standard exercise and nutrition information + up to five face-to-face motivational interviewing sessions PA specialists, registered dietician	Six m	IPAQ	Baseline and follow-up changes: IG: 198 ± 63 Met min/week walking CG: –145 ± 109 Met-min/week walking IG: 245 ± 104 Met min/week total PA CG: –122 ± 158 Met min/week total PA	The IG significantly increased walking and total PA when compared to the CG
Elley et al., 2003 [18]	5/10	878 sedentary participants. IG: 451 (301 women), 57.2 ± 10.8 years CG: 427 (281 women), 58.6 ± 11.5 years	A prompt card, stating the stage of change, + oral and written advice by GP in the consultation + at least three telephone calls (lasting 10–20 min) over the next three months GP and practice nurse	Three m	Three-month PA recall questionnaire	Mean changes (95% CI): IG: 54.6 (41.4–68.4) min/week CG: 16.8 (6.0–32.4) min/week	The IG showed greater increase in PA during leisure time and total energy expenditure than the CG

CAD: coronary artery disease; CG: control group; CHAMPS: community healthy activities model programme for seniors; CVDs: cardiovascular diseases; ES: exercise scientist group; GP: general practitioner; IG: intervention group; IPAQ: international physical activity questionnaire; HeiQ: health education impact questionnaire; n/a: not available; PA: physical activity; P: pedometer; m: months; SD: standard deviation; SQUASH: short questionnaire to assess health enhancing physical activity; T2DM: type 2 diabetes mellitus; w: weeks; FTA: few touch application; FTA HC: few touch application with health counselling.

The studies had a mean methodological quality score of 7.2 out of 10, ranging from 5 to 9 on the PEDro scale (Table 1). Lack of blinding was the most evident methodological flaw in the studies. Failure to conceal allocation was another general methodological limitation.

The 15 studies enrolled a total of 6727 participants; the sample size ranged from 74 to 878 adults [17,18]. Among these studies, seven enrolled adults eligible for CVDs risk assessment or presenting at least one cardiovascular risk factor [16,19–23], one enrolled adults at risk of diabetes or CVDs, two studies encompassed sedentary participants, three included participants with diabetes, or adults with hypertension or diabetes [8], and another study included family members of patients with coronary artery disease [18,24,26–29].

The health education interventions were delivered in several ways, including face-to-face sessions [19–22,26], by telephone [8,9,28], group sessions and a combination of face-to-face, telephone, and group sessions [16–18,23,24–27,29]. The method most used was face-to-face sessions plus telephone. There were differences between the frequency of sessions and the length of the interventions. Interventions varied in length from six hours to 12 months [8,19,24,26–29].

Regarding the methodology used to assess PA, the great majority of studies used a questionnaire. Only one study used an objective measure (accelerometry) together with the Norfolk Physical Activity Questionnaire [27]. The international physical activity questionnaire (IPAQ) and the community healthy activities model programme for seniors (CHAMPS) questionnaire were used in three studies each [8,21,22,23,26]. The other eight studies used different questionnaires (Table 1).

### Lifestyle outcomes

Behavioural outcomes included PA, diet, alcohol consumption and smoking status. Significant improvements in the PA levels of the interventional group (IG) compared with the control group (CG) were reported in eight studies (Table 1) [8,18,20–22,25,26,29]. Armit et al. showed an increase in PA at weeks 12 and 24 with no significant group differences; nonetheless, at week 24, the group receiving behaviour change advice plus a pedometer was more likely to report meeting PA guidelines than the CG group [25]. Additionally, three studies found a significant increase in the PA levels of both groups. In these studies, the CG also received an intervention, i.e. they received more than the usual care [19,24,27]. In the Cochrane et al. study the CG received the NHS health check plus the usual general practitioner care [19]; Lakerveld et al. provided the CG with brochures with information and guidelines with regard to healthy PA levels, a healthy diet and, if relevant, smoking cessation [24]; in the study of Griffin et al. the CG received an intensive treatment which included among other features dietary counselling, more frequent contacts with the general practitioner and theory-based education [27]. Only four studies reported a lack of significant effects of the intervention on PA levels [9,17,23,28].

Dietary intake was reported in 10 studies [8,19–24,27–29]. Harris et al. reported a daily enhancement in the number of portions of fruit and vegetable consumed in the IG at six months, but showed no significant differences at 12 months [20]. Koelewijn-Van Loon et al. showed a significantly lower intake of fat in the IG and a significantly higher percentage of participants meeting the recommendations for vegetable intake than in the control group [23]. Hardcastle et al. described a lower fat intake at six and 18 months [22].

Smoking and alcohol consumption were measured in eight studies [16,19,20,23,24,26,27,29]. Only Davies et al. reported a decrease in smoking status in the intervention group at eight and 12 months follow-up compared with the control group [26].

### Cardiovascular risk outcomes

All studies used physiological outcomes to assess the effects of the health education interventions. They measured HbA1c [26], fasting blood glucose [20,29], blood pressure [19–22,25,26,29],, lipid profile [19–22,26,29], body mass index [9,19,20,29], waist circumference [9,19,20,29], body weight [19,20,21,22,26], heart score [23,26], Framingham score [9,18,19], and type 2 diabetes mellitus risk score [24].

Davies et al. showed reductions in body weight compared with control groups with a decrease of 3.1% at four months and 3.2% at 12 months [26]. The effects of health education interventions on waist circumference were significant in two studies and not significant in other two studies [9,19,20,29]. In some studies, risk factors, such as high cholesterol levels and blood pressure [19,20,25,29], did not improve during the study period [20,22,23,26,29]. However, other studies showed a significant decreased in blood pressure [9,21,22], total cholesterol and triglycerides after the intervention [9,21,22,26].

Regarding the heart score, Davies et al. reported a greater improvement in the IG in comparison to the CG [26]. Wister et al. also reported a decrease in the Framingham risk score of 3.1% [9] and Cochrane et al. reported a decrease of 2.98% [19].

## Discussion

### Main findings

Despite the differences between studies in the methods and instruments used to assess PA, the available evidence suggests that health education interventions are successful in the modification of PA levels in primary prevention. Despite the use of different indicators, in several reviewed studies where PA changes were statistically significant, the improvement ranged from 5% to 16% [19,20,22,27,29]. Moreover, another study reported *odds* for increasing PA in the intervention group of 3.82 [8], while in another study the *odds* to reach the PA recommendations was 2.39 [25]. Overall, the health education interventions seem to have positive repercussions on cardiovascular risk factors, mainly on lipid profile, blood pressure and cardiovascular risk score.

### Strengths and limitations

Most the studies included in this review showed high methodological quality and, with two exceptions [8,25], have conducted an a priori power analysis to determine the sample size. In the majority of the studies, PA was assessed using different questionnaires, which may have limited the observation of the real impact of the interventions. Self-reported PA measures have low sensitivity, high variance, are less accurate, and frequently overestimate the PA levels [30]. Moreover, some of the questionnaires do not stratify PA by intensity levels (light, moderate, vigorous or very vigorous), precluding the analysis of the PA by intensities. Objective data is needed to measure sedentary time and/or the time spent in sedentary behaviours (sitting, TV viewing, time in bed rest) because it has been questioned whether the accomplishment of the recommended amount of daily physical activity time at moderate-to-vigorous intensities is sufficient to overcome long periods of sedentary behaviours, and yet protect the individuals against cardiovascular risk factors [31].

Most studies in this review did not consider the influence of environmental factors such as seasonal changes in daily physical activity. This cannot be disregarded when studying the long-term effects of health education interventions in the modification of PA levels since it was already shown there are significant differences in the amount and patterns of PA between winter and other seasons [32,33].

One limitation of this review is potential publication bias, as it included only articles published in English.

### Interpretation

The studies that showed significant changes in PA have adopted face-to-face, at least one session, plus remote interventions and used motivational techniques for behavioural change such as motional interviewing sessions. Interventions incorporating cognitive behavioural strategies, including goal-setting, self-monitoring, face-to-face contacts, feedback and reinforcement are more likely to induce changes [34]. Despite assuming that the PA behaviour of the individuals might be influenced by these intervention characteristics, we cannot assume that this is the most effective delivery method because these common methodological traits were not substantially different from some of the studies not reporting changes in PA after the intervention.

In addition to the improvements in PA, several cardiovascular risk factors and risk score were positively change by the interventions, which make it clinically important. Indeed, even small but sustained lifestyle changes can substantially reduce cardiovascular risk, morbidity and mortality [34].

### Implications for further research

In most studies, the sample was mixed gender cohorts. Therefore, future studies should include sub-analysis by gender to ascertain whether between-gender differences exist. They should also include measures of the time spent at different PA intensities, namely at moderate-to-vigorous PA, and sedentary behaviours, because sedentary time has been consistently related to deleterious health outcomes [35]. Environmental factors should be monitored, and PA contexts (occupational, leisure-time physical activity) must be differentiated. Since motivation or self-determination for exercise is an intra-personal correlate or determinant for the behavioural changes [36], future studies should report motivation levels or states of self-determination at baseline and how these features change throughout the interventions. Furthermore, future studies should also report the effect sizes, thereby enabling the appreciation of the magnitude in the differences between groups, and therefore, how effective the interventions are.

## Conclusion

The current research provides evidence that health education interventions are successful in the modification of PA levels in primary prevention. The health education interventions seem to have also a positive impact on cardiovascular risk factors, mainly on lipid profile, blood pressure and cardiovascular risk score.

## Supplementary Material

PRISMA 2009 Checklist
